# The risk for developing neurological sufferances in Romanian HIV infected patients – predictive criteria

**DOI:** 10.1186/1471-2334-14-S4-P33

**Published:** 2014-05-29

**Authors:** Lucian Giubelan, Augustin Cupşa, Florentina Dumitrescu, Andreea Stoian, Irina Niculescu

**Affiliations:** 1 University of Medicine and Pharmacy, Craiova, Romania

## 

Objective: to establish a set of predictive criteria for the occurrence of neurological sufferances in a group of Romanian HIV infected patients (Px).

Retrospective study (January 1990 – January 2011): we have stratified data (CD4 count, HIV-RNA level, total duration of antiretroviral treatment (ART) and duration of a certain ART regimen) from 98 Px diagnosed with neurological sufferances. Chi2 test, odds ratio (OR), relative risk (RR) and Receiver-Operator-Curve (ROC) have been used for statistical reasons.

OR increases exponentially with the decrease of CD4 count (max. 37.68), total ART duration (max. 17.7) and duration of a certain regimen (max. 62.37) and linearly with the HIV-RNA level (max. 47.75). RR increases linearly for all the studied parameters (7.36, 3.73, 2.88 and 7.64 respectively). ROC analysis suggests that, for our group of Px, less than 178 CD4 cells/cmm (sensitivity = 58.2, specificity = 85.2, p<0.0001), more than 20,750 HIV-RNA copies/cmm (sensitivity = 72.2, specificity = 46.7, p value not statistically significant), a total ART duration of less than 41 months (sensitivity = 49.0, specificity = 77.6, p<0.0001) or less than 23.5 months for a specific ART regimen (sensitivity = 72.4, specificity = 63.2, p<0.0001) should be predictive for neurological sufferances.

CD4 count, HIV-RNA level, total ART duration and duration of a certain ART regimen could be used to predict the occurrence of neurological sufferances in Romanian HIV infected Px, however the best results (given the ROC sensitivity and specificity) are provided by the duration of a specific ART regimen.

